# Exon skipping induced by CRISPR-directed gene editing regulates the response to chemotherapy in non-small cell lung carcinoma cells

**DOI:** 10.1038/s41434-022-00324-7

**Published:** 2022-03-22

**Authors:** Kelly Banas, Shirin Modarai, Natalia Rivera-Torres, Byung-Chun Yoo, Pawel A. Bialk, Connor Barrett, Mona Batish, Eric B. Kmiec

**Affiliations:** 1 Gene Editing Institute, ChristianaCare, Newark, DE USA; 2 Department of Medical and Molecular Sciences, University of Delaware, Newark, DE USA

**Keywords:** Molecular biology, Non-small-cell lung cancer, Transcription

## Abstract

We have been developing CRISPR-directed gene editing as an augmentative therapy for the treatment of non-small cell lung carcinoma (NSCLC) by genetic disruption of Nuclear Factor Erythroid 2-Related Factor 2 (NRF2). NRF2 promotes tumor cell survival in response to therapeutic intervention and thus its disablement should restore or enhance effective drug action. Here, we report how NRF2 disruption leads to collateral damage in the form of CRISPR-mediated exon skipping. Heterogeneous populations of transcripts and truncated proteins produce a variable response to chemotherapy, dependent on which functional domain is missing. We identify and characterize predicted and unpredicted transcript populations and discover that several types of transcripts arise through exon skipping; wherein one or two *NRF2* exons are missing. In one specific case, the presence or absence of a single nucleotide determines whether an exon is skipped or not by reorganizing Exonic Splicing Enhancers (ESEs). We isolate and characterize the diversity of clones induced by CRISPR activity in a NSCLC tumor cell population, a critical and often overlooked genetic byproduct of this exciting technology. Finally, gRNAs must be designed with care to avoid altering gene expression patterns that can account for variable responses to solid tumor therapy.

## Introduction

The promise of genomic medicine is dependent on both discovering the genetic aberration and developing the therapeutic tools to alter it. For many years, the field was limited to discovery, however this changed dramatically with the discovery of CRISPR and its adaptation as a tool for human cells. We have proposed an innovative therapeutic approach to boost or restore chemo-sensitivity through precise genetic disruption of Nuclear Factor Erythroid 2-Related Factor 2 (NRF2), a master regulator of 100–200 target genes primarily involved in cellular responses to oxidative/electrophilic stress [1, 2]. Increased expression of NFR2 promotes cell proliferation, inhibits apoptosis, and upregulates downstream target genes, which encode drug-metabolizing enzymes, drug transporters, and stress response proteins [3]. The strategy employs Clustered Regularly Interspaced Short Palindromic Repeats (CRISPR)-directed gene editing and leads to total or functional disruption of targeted human genes [4, 5].

We previously reported that functional knockout of *NRF2* in chemo-resistant lung cancer cells enhanced the activity of cisplatin, carboplatin, and vinorelbine in both cell culture and xenograft mouse models [6, 7]. The resistance to anticancer drugs is exacerbated by the fact that chemotherapy amplifies transcription of NRF2 target genes often triggering a cytoprotective response in cancer cells. Increased NRF2 expression accounts for chemoresistance, particularly in non-small cell lung carcinoma [2, 8]. In contrast to the oncogenic role of NRF2, this protein has a dual function as it may help to confer cyto-protection in immune cells [9].

While this approach seems straightforward, we are moving cautiously, considering the molecular gymnastics that can take place on human chromosomes as a function of double-stranded cleavage, including breaks generated by CRISPR/Cas activity. As such, we have been focused on investigating the *totality* of genetic outcomes of *NRF2* disruption because we now know that CRISPR/Cas activity can occasionally lead to unwarranted and often complex genetic changes [10–18]. Among the most impactful outcomes of chromosomal rearrangement is the alteration in expression patterns of targeted, and nontargeted, genes.

In this manuscript, we investigate the consequences of *NRF2* disruption, focusing on genomic rearrangement as collateral damage resulting from inherent CRISPR/Cas activity. We identify and characterize predicted and altered transcript populations and we discover that altered transcripts arise from the process of exon skipping; wherein transcripts missing one or two *NRF2* exons are generated in the mature transcript population. The molecular basis of these altered transcripts is disruption of Exonic Splicing Enhancer (ESE) sequences [19], an outcome we consider to be as potentially harmful as the well-publicized off-site mutagenesis. In fact, one could argue that exon skipping is more important since we are observing a direct impact of targeted gene expression. Importantly, we discover that exon skipping is not simply a genetic abnormality, but in fact, can regulate the functionality of a tumor cell in its response to chemotherapy. In some cases, the response is governed by which of the protein domains remains intact and which is absent. We suggest that detailed molecular analyses of all the genetic consequences of CRISPR-directed gene editing, at the genotypic and phenotypic levels, should become the norm not the exception when investigators advance clinical development programs for the treatment of both liquid and solid tumors.

## Materials and methods

### Cell line and culture conditions

Human lung adenocarcinoma A549 cells (#CCL-185, passage number 1-12) and human lung squamous cell carcinoma NCI-H1703 [H1703] cells (#CRL-5889, passage number 1-12) were purchased from ATCC (Manassas, VA, USA). A549 clonal cell lines, 1-40 and 2-11, were obtained from Bialk et al [6]. Cells were thawed, according to the manufacturer’s protocol. A549 cells were grown in F-12K medium (ATCC) supplemented with 10% fetal bovine serum (FBS) (ATCC). NCI-H1703 cells were grown in RPMI 1640 medium (ATCC) supplemented with 10% fetal bovine serum (FBS) (ATCC). Both cell lines were grown at 37 °C in 5% CO_2_. Cell lines were tested for Mycoplasma upon thawing and before use in experiments using the MycoScope PCR Mycoplasma detection kit (Genlantis, Cat. MY01100).

### CRISPR/Cas9 design and assembly

The *NRF2* gene-coding sequence was entered into Benchling (https://benchling.com) and the following gRNAs were selected for targeting exon 2: (1) 5′-TATTTGACTTCAGTCAGCGA-3′, (4) 5′-TGGAGGCAAGATATAGATCT-3′. Based on the gRNA design, synthetic single gRNAs were ordered from Synthego (Menlo Park, California, USA). Recombinant spCas9 protein was purchased from Integrated DNA Technologies (Coralville, Iowa, USA) (62 μM stock solution). sgRNA and SpCas9 protein were mixed at a 5:1 ratio (250:50 pmol) and set to incubate at room temperature for 15 min before transfections. To target exon 4 of *NRF2*, the following gRNAs, (2) 5′-TCGATGTGACCGGGAATATCAGG-3′ and (3) 5′-TGATTTAGACGGTATGCAAC-3′, were designed and used as plasmid constructs (pX458), as previously described [6].

### Transfection and clonal isolation

A549 and NCI-H1703 cells were seeded 48 h prior to transfection and allowed to reach 60–80% confluency. On the day of the transfection, cells were harvested by trypsinization and washed twice with 1× PBS (-/-). Cells were resuspended at a concentration of 3 × 10^5^ cells/20 μL in SF/supplement solution and 5 μL of RNP complex was added to each sample. Lonza program CM-130 was used and after 15 min of rest, cells were transferred to six-well plates for 48 h prior to sorting. Transfected A549 cells were sorted into 96-well plates with a FACS Aria II flow cytometer (BD Biosciences, Franklin Lakes, NJ, USA). Clones were expanded and transferred to larger plates as the individual clones reached confluence. A549 clonal cell lines, listed in Fig. 3, were transfected and clonally expanded as previously described [6]. Both *NRF2* targeting pX458 constructs were simultaneously electroporated into A549 cells, single-cell sorted, and clonally expanded.

### Gene editing analysis

Cellular genomic DNA was isolated from each clonal cell line using the DNeasy Blood and Tissue Kit (Qiagen, Cat. 69506). The region surrounding the CRISPR target site was PCR amplified using the Q5 High-Fidelity 2X Master Mix (New England BioLabs, Cat. M0492) (Exon 2 – FWD primer 5′ CACCATCAACAGTGGCATAATGTGAA 3′, REV primer 5′ AACTCAGGTTAGGTACTGAACTCATCA 3′) (Exon 4 – FWD primer 5′ GTAGTGGTGCCTTAGAGCTTACTCATCC 3′, REV primer 5′ CTAGCATGGGCAGTACTCATGACTAAG 3′). The PCR reaction was purified using the QIAquick PCR Purification Kit (Qiagen, Cat. 28106) and Big Dye Terminator PCR was performed using Big Dye Terminator v3.1 (Thermofisher). PCR products were purified once more using the Big Dye Xterminator kit (Thermofisher) and then sequenced using the SeqStudio Genetic Analyzer (Applied Biosystems). Clonal allelic analysis was conducted using the software program, DECODR, available at https://decodr.org/analyze. The DECODR deconvolution algorithm is written in Python 3.6.8. All modules and libraries implemented within the software are either open-source or custom-written. Input files consist of one control file per analysis (plain text, FASTA or. ab1) and any number of experimental files (FASTA or. ab1), along with up to 2 CRISPR guide sequences (plain text), an optional homology donor DNA sequence (plain text), and a nuclease (Cas9 or Cas12a). If text-based files are provided, they are interpreted as chromatograms with identical peak heights for each position.

### Transcript analysis

Total RNA was isolated from each clonal cell line using TRIzol reagent (Thermofisher Scientific, cat. 15596026) and the Pure Link RNA mini-Kit (Thermofisher Scientific, cat. 12183018A). Reverse transcription of total RNA was conducted using the High Capacity RNA-to-cDNA kit (Thermofisher Scientific, cat. 4387406). The cDNA was used as the template for standard PCR and amplification of NRF2 transcripts using the Q5 High-Fidelity 2X Master Mix. The following sets of PCR primers were used: FWD 5′ CCAACACACGGTCCACAGCTCAT 3′, REV 5′ GAAGACTGGGCTCTCGATGTG 3′; FWD 5′ GCGCAGACATTCCCGTTTGTAGA 3′, REV 5′ GAGGATGCTGCTGAAGGAATCCTC 3′; FWD 5′ CCAACACACGGTCCACAGCTCAT 3′, REV 5′ GAGGATGCTGCTGAAGGAATCCTC 3′. The PCR reaction was purified using the QIAquick PCR Purification Kit and Big Dye Terminator PCR was performed using Big Dye Terminator v3.1. PCR products were purified once more using the Big Dye Xterminator kit and then sequenced using the SeqStudio Genetic Analyzer.

### Western blot analysis

A549 and H1703 clonal cell lines were harvested and the western blot was carried out as previously described [6]. Primary antibody incubation was performed overnight on a shaker at 4 °C for NRF2 (1:1,000, Abcam ab62352), and GAPDH (1:10,000, Abcam ab8245), and secondary antibody (Jackson ImmunoResearch Laboratories, West Grove, PA, USA) incubations were all done 1 h at room temperature at a 1:10,000 dilution. The protein bands were visualized via chemiluminescence using a SuperSignal West Dura Extended Duration ECL (Pierce) and detected on the LI-COR Odyssey FC. Parental A549 cells were used as a control shown in the first lane after the protein ladder, of each western blot image.

### Single-molecule fluorescent in situ hybridization (FISH)

Probe sets of small 20nt probes complementary to exon 2 (13 probes), exon 3 (4 probes), and exon 5 (48 probes) were designed with 3′ amino modifications. The probes for exon 2 and exon 3 were labeled en mass with cy5 and the probes for exon 5 were labeled with Alexa 594. The labeled probes were then purified using a reverse-phase HPLC. A549 parental and clonal cells were grown on glass coverslips. The cells were fixed using 4% paraformaldehyde for 10 min, permeabilized with 70% ethanol at 4 °C, and hybridized overnight at 37 °C with labeled probes. The coverslips were washed to remove unbound probes, stained with DAPI, and mounted. [20] The coverslips were imaged using an inverted Nikon TiE florescence microscope equipped with a CCD Princeton PIXIS 1024b camera. The images were acquired using Metamorph software. The images were analyzed using custom written programs in MATLAB (Mathworks Inc.). The quantification programs are available upon request. Statistical analysis was conducted using Student’s *t* test between the A549 clones and A549 wildtype cells.

### MTS cell proliferation assay

Cell viability after drug exposure was evaluated using the CellTiter 96 Aqueous Non-Radioactive Cell Proliferation Assay (Promega, Madison, WI). A549 cell lines were plated in quadruplicate at 2 × 10^3^ cells per well and allowed to culture for 24 h. The cells were then treated with cisplatin for three days. The cell media was aspirated, the cells washed with PBS, then exposed to the MTS reagent for 3 h. After 3 h of MTS bio-reduction by proliferating cells, the formazan product’s absorbance was measured using a 450 nm filter on an Infinite 2000 PRO microplate reader (Tecan, Mannadorf, Switzerland). Each experiment was conducted twice, and the data was normalized and averaged for each clone at each concentration. The standard error of the mean was calculated using the normalized value of all eight data points for each clone at each concentration. Statistical analysis was performed using Graphpad Prism 9 (San Diego, CA, USA) and statistical significance was defined as *P* < 0.05. For comparisons among multiple groups, two-way ANOVA followed by Tukey’s multiple comparison test was performed. IC50 values were derived from linear regression analysis of the data in the response curve.

### CRISPinatoR ESE analysis

For ESE analysis, the website http://crispinator.com/ko/ was used to identify the location of ESEs along the coding region of the *NRF2* (searched as *NFE2L2*). All original search criteria were left as is.

## Results

**Fig. 1:**
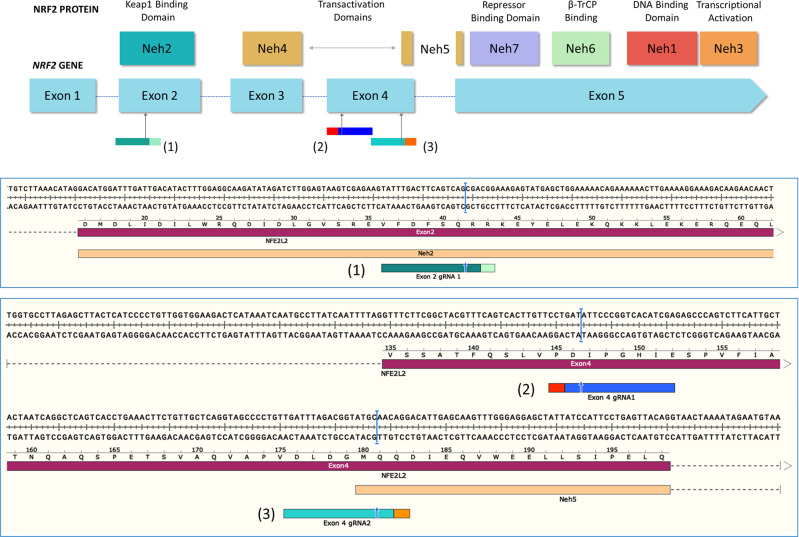
CRISPR design and *NRF2* sequence target. The top panel displays the structural domains of the NRF2 protein aligned to the exons of the *NRF2* gene. Three guide RNAs were designed to cleave within exon 2 and exon 4 of the gene. The top panel provides a schematic diagram of the cleavage sites of each guide RNA in relation to the gene and protein. The bottom panel presents sequence alignment and actual cleavage sites of each guide RNA. Guide RNA (1) was designed to target the beginning of exon 2 whereas guide RNA (2) and (3) were designed to target the beginning and end of exon 4, respectively.

Previous work from our laboratory established that the genetic disruption of *NRF2* in A549 cells, a cell line derived from a lung adenocarcinoma [6, 7, 21, 22], resulted in an increase in chemosensitivity [6]. In this work, we extend those studies using a series of CRISPR/Cas complexes designed to cleave and disrupt *NRF2* at various sites within the gene. Figure 1 provides an overview of our broad-based CRISPR/Cas9 gene-editing strategy. A single guide RNA (gRNA) designated (1), was used to target exon 2, which encodes the Neh2 domain of the protein. A separate pair of gRNAs designated (2) and (3) was used to target exon 4, which encodes the first half of the Neh5 domain. A dual gRNA approach was also used where both gRNAs (2) and (3) were simultaneously transfected to generate large fragment deletions within exon 4. By targeting two nonadjacent exons of *NRF2*, we can analyze the consequence of molecular rearrangements induced by CRISPR/Cas activity in A549 cells.

### CRISPR-directed genetic engineering of the NRF2 gene using single guide RNAs induces exon skipping

**Fig. 2:**
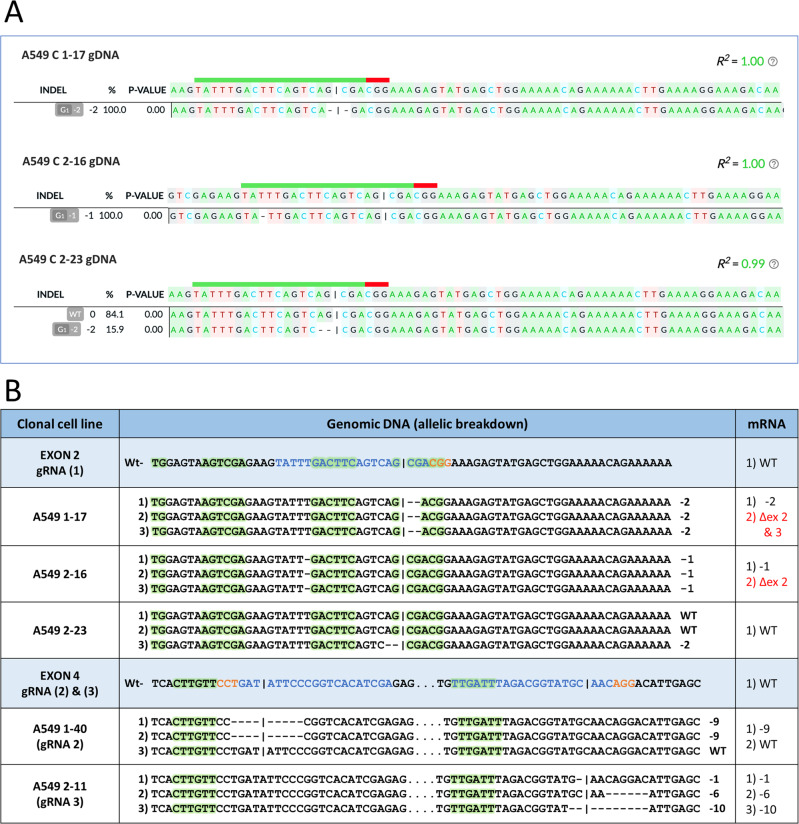
Genomic and transcript analyses of A549 clonal cell lines created by CRISPR targeting. **A** Genomic DNA from clonal cell lines, 1-17, 2-16 and 2-23, was isolated and amplified across exon 2 of the *NRF2* gene. Amplicons were sanger sequenced and analyzed for indels at the CRISPR target site. Raw sequence files were aligned using the software program, DECODR, to display the *NRF2* allele-specific indel pattern (listed as INDEL and %). **B** The first column lists the cell line and clonal identification number with the associated guide RNA (blue) used to target *NRF2*. The second column lists the genomic *NRF2* sequence of each clone and the allele-specific indel patterns with the normal genomic sequence are listed before each set. The guide RNA sequence is depicted in blue and the PAM in orange with the cleavage site represented by the vertical line. Exonic splicing enhancers are highlighted in green within the second column. The third column lists the population of mRNA transcripts of each clonal cell population.

The genetic analysis of the knockout of *NRF2* - specifically the genotype of clones 1-17, 2-16, and 2-23, is presented in Fig. 2A. These clones were created by targeting exon 2 with a single gRNA (gRNA (1)). The genomic indels for each clone were deconvoluted by the deconvolution software program DECODR [23] through paired-end analysis, which utilizes both forward and reverse sequencing of each clone. DECODR presents the indel distribution of each clone from raw sequence data and the percentages correspond to the indels for each allele. In this case, most of the clones present with three alleles, as the A549 cell line is known to be hypo-triploid [24]. The 100% (e.g., Clone 1–17, Clone 2–16) indicates all alleles contain that indel listed. The 84/16% (e.g., Clone 2-23) indicates 84% of the genomic DNA is wildtype with about 16% containing the two base pair deletion. This roughly corresponds to two wildtype alleles and one mutant allele. Our genetic analysis indicates that clone 1-17 contains a two-base pair deletion at the target site across all three alleles causing a frameshift mutation that forms stop codons downstream. Clone 2–16 bears a single base pair deletion upstream of the target site also inducing a frameshift that forms stop codons further downstream. In contrast, clone 2-23 contains a single allelic disruption through a two-base pair deletion at the target site causing a frameshift mutation and forming stop codons downstream. The other two alleles of the *NRF2* gene in clone 2-23 are wild type.

We sought to examine if the genetic disruption of *NRF2* would influence the types of mRNA populations in the targeted cell (Fig. 2B). The first column lists the clonal identification number with the associated gRNA used to target *NRF2*. The second column lists the genomic *NRF2* sequence of each clone and the allele-specific indel patterns induced by the gRNA listed. The normal genomic sequence is listed before each set of clones with a depiction of the gRNA sequence in blue, the PAM in orange, and the cleavage site represented by the vertical line. The third column lists the population of mRNA transcripts arising from each clonal cell line generated by the action of CRISPR-directed gene editing. For example, an indel (i.e., −2) listed in the third column indicates that this indel, initially identified by genomic sequencing, is also apparent in the mRNA transcript population. In most of the clonal cell lines, the second transcript population may arise from an exon skipping event, likely induced by ESEs [19], which are highlighted in green. The abundance of truncated transcripts produced as a function of CRISPR-directed gene editing led us to examine the DNA sequences proximal to the targeted site. CRISPinatoR, developed by Tuladhar et al., is a web-based sgRNA design platform for CRISPR/Cas9 that searches for ESE sites that enable transcript modification at the genomic level. Our suspicion was that CRISPR/Cas9 activity modified some regulatory sequences that subsequently would enable or eliminate exon skipping activity within and surrounding the *NRF2* gene (Supplementary Fig. 1). Even though the phenomenon of exon skipping has been previously reported, we felt it was important to demonstrate genetic rearrangement vis-à-vis exon skipping in a separate cell line. Therefore, we were able to confirm the presence of similar rearrangements in the NRF2 gene in a separate cell line, H1703, with different gRNA designs (Supplementary Fig. 2A). Utilizing different gRNAs to achieve exon skipping, associated with CRISPR-directed gene editing, demonstrates the broad-based nature of this metabolic activity.

Clones 1-17 and 2-16 each harbor two different transcript populations, the first reflecting the CRISPR-induced alterations present in the genomic DNA and the second population showing exon skipping. Interestingly, despite containing a genomic indel on a single allele in addition to two wildtype alleles at the *NRF2* locus, clone 2-23 contains only a single wildtype transcript population. The cleavage site of gRNA (1) falls between the first two nucleotides (G–C) of the fifth ESE site in exon 2. The location of each ESE relative to the CRISPR target site seems to play a key role in the exon skipping events.

In the lower section of Fig. 2B, we present two clonal cell lines (1-40 and 2-11) generated by targeting exon 4 of *NRF2* using two separate gRNAs, as previously described [6]. Genetic analysis reveals distinct indel patterns at the target site in each clone. Clone 1-40 bears two alleles with the same indel pattern and a single wildtype allele, whereas clone 2–11 contains three unique indels at the target site. Interestingly, when analyzing the transcript populations in both clones, we only detected transcripts that reflect the alterations seen in the genomic DNA, with no exon skipping. It appears when the initial CRISPR/Cas9 cleavage activity occurs distal to the sequences identified as ESEs [19], the integrity of these cis-acting sequence elements is maintained allowing them to function properly.

### CRISPR-directed genetic engineering of the NRF2 gene using dual guide RNAs induces exon skipping

**Fig. 3:**
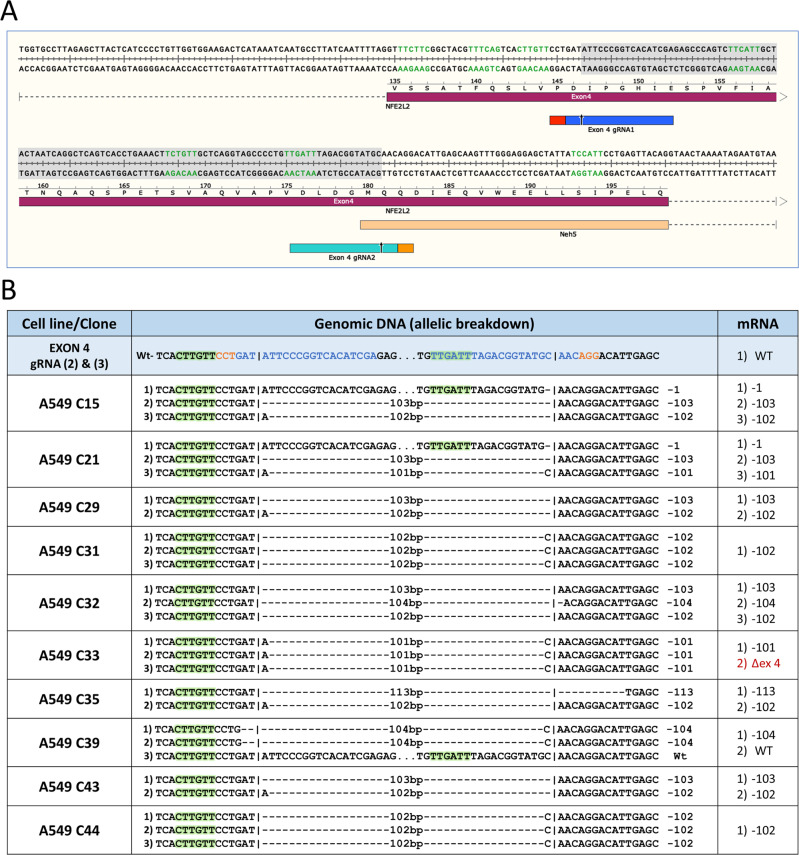
Genomic and transcript analyses of A549 clonal cell lines created by dual CRISPR targeting. **A** Schematic diagram of the actual cleavage sites of guide RNA (2) and (3) and the resulting 103 base pair fragment deletion caused by using the dual guide RNA approach. The exon splicing enhancers are shown in green. **B** The first column lists the cell line and clonal identification number with the associated guide RNA used to target *NRF2*. The second column lists the genomic *NRF2* sequence of each clone and the allele-specific indel patterns with the normal genomic sequence is listed before each set. The guide RNA sequence is depicted in blue and the PAM in orange with the cleavage site represented by the vertical line. Exonic splicing enhancers are highlighted in green within the second column. The third column lists the population of mRNA transcripts of each clonal cell population.

To evaluate the universality of the exon skipping phenomena, we utilized a dual system of gRNA (2) and gRNA (3) (see Fig. 3A) which aims to disrupt exon 4 thereby establishing another family of clonal variants (Fig. 3B). The dual guide RNA approach was designed to remove a 103 base pair fragment from the middle of exon 4. Of thirty-two clonal cell lines, ten were expanded and further characterized. Each clone reveals a diversity of genomic signatures including the loss of three ESE sites. Of the ten clones characterized, however, only clone 33 displayed exon skipping of exon 4. Interestingly, clonal cell lines 31, 33, and 44 differ by only one base pair yet clones 31 and 44 do not produce a population of transcripts with exon skipping. Both clones 33 and 21 contain 101 bp deletions, yet only clone 33 exhibits exon skipping. Taken together, our data suggest the outcomes of genetic knockout, even at the single nucleotide level due to nonhomologous end-joining activity (resection) can induce or suppress exon skipping.

### Multiple transcript populations generated by CRISPR-directed gene editing are also revealed at the protein level

**Fig. 4:**
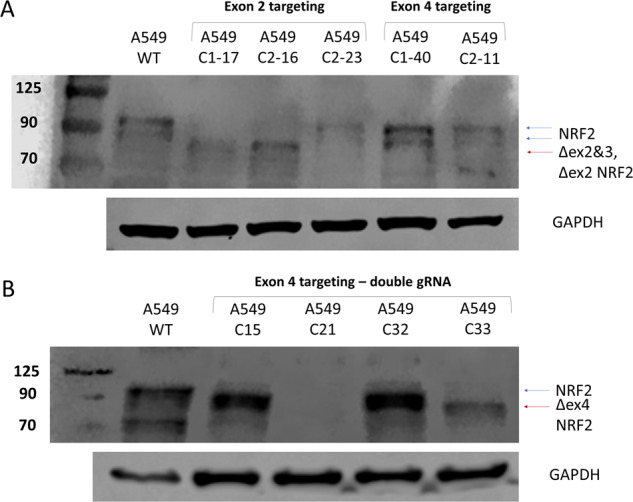
Representative western blot analysis of CRISPR-engineered A549 clonal cell lines. Clonal cells were harvested for western blot analysis using an antibody directed against NRF2 and GAPDH was used as a loading control. **A** This image displays the detection of NRF2 protein from A549 clonal cell lines targeted with a single CRISPR guide RNA in both exon 2 and exon 4, as marked. Lanes 2-6 contain whole cell lysates from NRF2 modified clonal cell lines. **B** This image displays the detection of NRF2 protein from A549 clonal cell lines targeted with two CRISPR guide RNAs in exon 4.

Since we mapped the genetic outcomes of CRISPR-directed gene editing at the genomic and transcript level, we sought to visualize exon skipping activity at the protein level. Western blot analysis of NRF2 in various cell lines has been particularly challenging because of differences in the predicted and actual molecular weight of NRF2 [25, 26]. Nevertheless, we were able to detect wild-type and modified NRF2 using a recombinant anti-NRF2 antibody purchased from Abcam (Cambridge, England) in both the A549 (Fig. 4) and NCI-H1703 cell line (Supplementary Fig. 2B). As shown in Fig. 4A, wildtype NRF2 (A549 cells) is visible, migrating between 95 kDa to 110 kDa, consistent with previous observations [26]. Two bands appear at ~90 kDa, which reflect what has been widely described as nuclear (top band) and cytoplasmic NRF2 (lower band) [25, 27–29]. Lane 2 contains whole cell lysate from clone 1-17, the clonal cell line which contains a population of transcripts with exon 2 and 3 skipping as well as transcripts with the two base pair deletion (see Fig. 2). As predicted, there is no visible protein migrating at 90 kDa because the transcript does not produce a protein, however, the band migrating at ~75 kDa represents the shortened transcript. In lane 3, the 90 kDa band is absent, again, indicating that the transcript with the single base pair deletion fails to produce a protein; the lower band seen in this lane reflects the exon 2 skipped transcript that we identified as part of the mRNA transcript population. In the fourth lane, whole cell lysate from clone 2-23 reveals a faint but visible band at 90 kDa and an associated band below it, like the first lane with A549 parental cells. As listed in Fig. 2, clone 2-23 harbors two wildtype alleles and a single mutated allele; hence, we expect wildtype protein to be produced. The final two lanes represent whole cell lysates from clone 1-40 and clone 2-11, respectively, which were created by targeting exon 4. In the fifth lane, clone 1-40 produces two visible bands at 90 kDa, which reflects the population of mRNA that is detected in the clonal cell line. This clone contains a wildtype allele along with two alleles with a 9-base pair deletion. In the sixth lane, whole-cell lysate from clone 2-11 reveals two bands at 90 kDa that are less visible than in clone 1-40, which corresponds with frameshifting indels (-1, -10) on two alleles and a non-frameshifting (-6) indel on the third allele, which is likely the transcript driving protein expression.

Further protein analysis of the genetically engineered clonal isolates is presented in Fig. 4B. The first lane, after the protein ladder, reflects the appearance of wildtype NRF2 (95 kDa) generated from A549 parental whole cell lysate. In the second lane is whole cell lysate from clone 15 with a visible band at 90 kDa. This clone contains a 1, 103, 102 base pair deletion in the genomic DNA which is reflected in the mRNA transcript. The 1 and 103 base pair deletion is frameshifting and is likely being degraded. However, the 102 base pair deletion is in-frame and most likely being transcribed into mutant protein that is being visualized in the western blot. The 102 base pair deletion is a loss of 34 amino acids which would result in protein about 4 kDa smaller in size. The same concept applies to the fourth lane which contains whole cell lysate of Clone 32. Clone 32 contains a 103, 104, and 102 base pair deletion seen in the genomic DNA and reflected in the mRNA transcript. The 103 and 104 base pair deletion is frameshifting and again likely being degraded, whereas the 102-base pair, like in clone 15, is in frame and being expressed as a mutant protein. In the third lane is whole cell lysate from clone 21 which contains a 1, 103, and 101 bp deletion in the gDNA and is reflected in the mRNA. In this instance, all three indels induce frameshifting and likely the reason for the complete knockout of NRF2. In the fifth lane is whole cell lysate for clone 33 which contains a homozygous 101 base pair deletion in the genomic DNA leading to exon 4 skipping in the mRNA. Since the 101 base pair deletion is frameshifting, the protein that is being picked up in the western blot is the exon 4 skipped protein. Exon 4 skipping is a loss of 192 base pair or 64 amino acids resulting in a protein about 7 kDa smaller in size. Based on the protein expression pattern in these four clones, each clonal cell line produces allele-specific protein (summarized in Supplementary Fig. 3).

### Confirmation of exon skipping transcripts driving altered protein expression

**Fig. 5:**
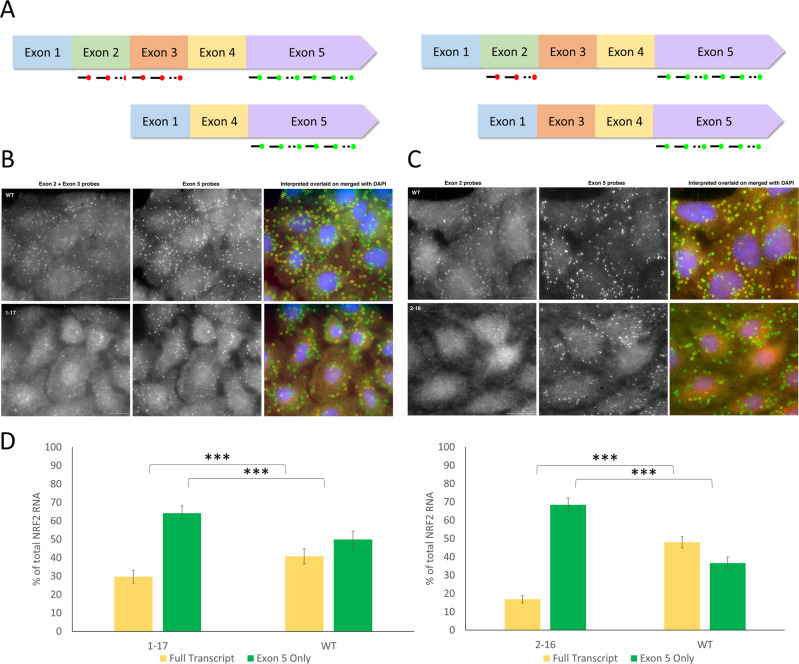
Detection of altered NRF2 transcripts by smFISH imaging. **A** Schematic diagram of the hybridization of labeled RNA probes to wildtype and altered NRF2 transcripts and the resulting fluorescence signal. **B** Representative images from A549 clone 1-17 cells labeled with probes for exon 2 and exon 3 labeled with cy5 and exon 5 labeled with Alexa 594. **C** Representative images from A549 clone 2-16 cells labeled with probes exon 2 labeled with cy5 and exon 5 labeled with Alexa 594. The gray-scale images in the two panels are merged images (z-stacks) from each fluorescence color channel followed by the merged image with Exon 2 and Exon 3 or just exon 2 pseudo colored as red, and the exon 5 signal pseudo colored as green. The images are merged with DAPI staining shown in blue and overlaid with circles obtained after analysis using custom-written program in MATLAB image processing software. **D** Quantification of the smFISH data after analysis of at least 100 cells of each type. The error bars indicate 95% confidence interval. The scale bar is 5 µm. ***P < 0.001.

As shown in Figs. 2 and 3, after CRISPR alterations of the *NRF2* gene, many of the clonal cell lines presented with alternatively spliced NRF2 transcripts. This was confirmed by cDNA sequencing, which showed two transcript populations—one reflecting exon skipping and the other reflecting the indel pattern noted in the genomic DNA. In contrast to the cDNA sequencing results, western blot analysis presumptively showed that only exon skipped isoforms are being expressed in these clonal cell lines. To confirm the expression and the cellular localization of these altered isoforms, we used single-molecule fluorescence in situ hybridization (smFISH) to visualize the transcripts being made in these clonal cell lines (A549 clone 1-17 and 2-16) as compared to the A549 parental cell line (WT) (Fig. 5). RNA probes were designed for exons 2, 3, and 5 of NRF2 and were subsequently labeled with different fluorescent tags to be able to differentiate between altered and full-length transcripts. The RNA probes specific for exon 5 were labeled with Alexa 594. Since exon 5 is present in all the transcript populations identified, labeling exon 5 provides a universal snapshot of the transcripts present. To distinguish between the exon skipped transcripts, probes for exon 2 and exon 3 were labeled with cy5. As shown in the schematic diagram of Fig. 5A, colocalization of both fluorescent probes would result in a merged fluorescent signal (yellow) and would indicate the presence of a full-length transcript. Transcripts lacking exons 2 or 3 would only bind the Alexa 594-labeled probes designed for exon 5, resulting in a single fluorescent signal (depicted as green).

Figure 5B and C depicts representative images from smFISH of both A549 clone 1-17 and clone 2-16. Figure 5B depicts smFISH comparing A549 parental and A549 clone 1-17 cells. These clonal cells contain two transcript populations—one reflecting the two base pair deletion seen in the genomic DNA and the other reflecting exon 2 and 3 skipping. By using smFISH, we can visualize and quantitate the transcripts being produced within the cell. As confirmed by cDNA sequencing, clone 1-17 produces “full-length” NRF2 transcripts (full length—yellow signal); however, the predominant transcript population visualized in these cells is exon skipped transcripts (Exon 5- green signal) (Fig. 5D). Figure 5C depicts smFISH comparing A549 parental and A549 clone 2-16 cells. These clonal cells also contain two transcript populations—one reflecting the one base pair deletion seen in the genomic DNA and the other reflecting exon 2 skipping. Like with clone 1-17, clone 2-16 also produces “full-length” NRF2 transcripts (yellow signal) however, the predominant transcript population in these cells is exon skipped transcripts (exon 5 total – green signal) (Fig. 5D). Based on the data presented in Fig. 5, we can confirm that in both clonal cell lines, the altered transcript is driving protein expression and influencing downstream pathways.

### Exon skipping induced by CRISPR-directed genetic engineering influences functional activity

The discovery and characterization of transcript populations in CRISPR-directed NRF2 knockout cell lines led us to ask whether such molecular change at the DNA and RNA level would affect the function of NRF2. One of its key functions is to protect cells against excess stress by activating genes involved in cytoprotective pathways. As such, NRF2 enables resistance to chemotherapy, and therefore disabling NRF2 should reduce resistance and elevate chemo-sensitivity [6, 7, 21].

**Fig. 6:**
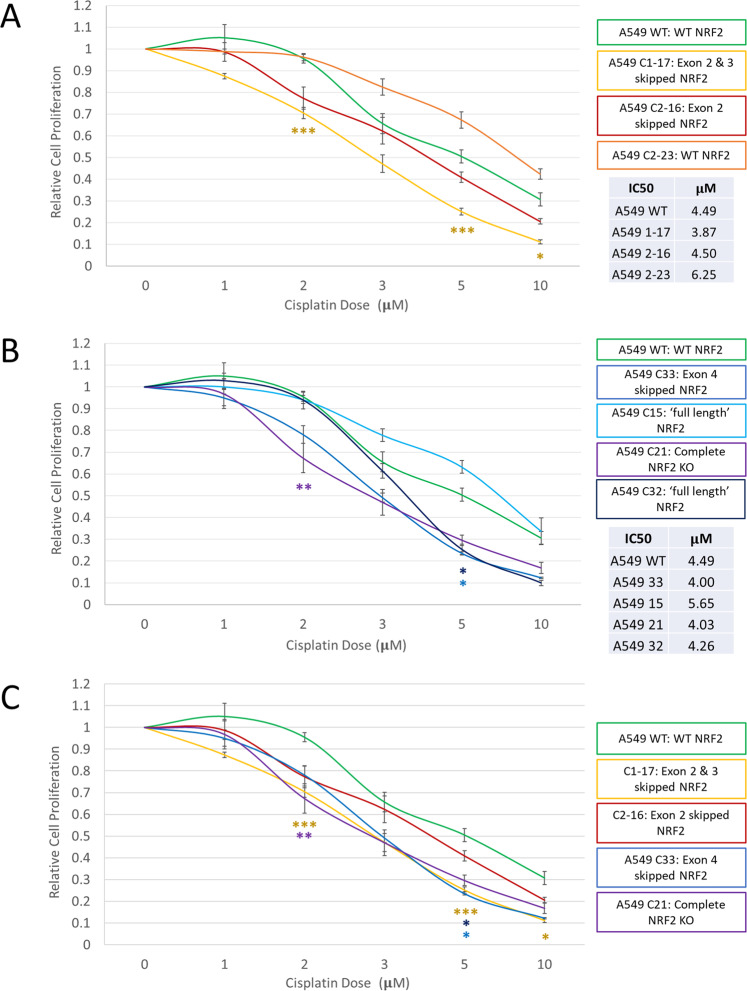
Proliferation capacity of wild type and NRF2 modified A549 clonal cell lines in response to cisplatin treatment. Proliferation was measured via bioreduction of MTS to a formazan product. Cells were treated with increasing concentrations of cisplatin for 72 h then evaluated for cell proliferation. The average relative proliferation of cells in response to cisplatin is graphed above. Exon 2 modified clonal cells are presented in **A** and exon 4 modified clonal cells are presented in **B**. **C** Data compiled from previous graphs for comparison of clonal cell lines with altered NRF2 expression. Error bars represent ±SEM, *n* = 8. *P < 0.05, **P < 0.01, ***P < 0.001 vs. A549 WT cells with the same treatment.

To evaluate changes in chemoresistance in a variety of clonal cell lines, we utilized the MTS viability assay, a colorimetric method for measuring metabolically active and viable cells in proliferation, cytotoxicity, and chemosensitivity assay. Figure 6A displays the relative cell proliferation of A549s and the derivative clones, 1-17, 2-16, and 2-23, when treated with various concentrations of cisplatin. Two separate experiments were conducted using each clone in quadruplet for a total of eight data points collected for each concentration of each clone.

On the genomic level, clone 1-17 contains a two-base pair deletion at the CRISPR cleavage site across all alleles to produce a mixed population of transcripts—one with the two base pair deletion and the other with exon 2 and 3 skipped. As confirmed through smFISH and western blot analysis, the altered transcript and isoform are driving phenotypic response to cisplatin treatment. Exon 2 and 3 skipping causes the loss of the functional Neh2 and Neh4 domain, respectively. The loss of these domains but not the Neh4 domain is impairing the functionality of NRF2, therefore increasing chemosensitivity of these cells as compared to the A549 parental cells which contain wildtype NRF2.

Clone 2-16 contains a single base pair deletion across all three alleles, 13 base pairs upstream of the CRISPR cleavage site. This single base pair deletion also leads to a mixed population of transcripts—one with the single base pair deletion and the other with exon 2 skipped. Also as confirmed through smFISH and western blot analysis, the altered transcript and isoform are driving the phenotypic response to cisplatin treatment. However, as seen in the graph, the response of clone 2-16 to cisplatin treatment is like that of the parental cell line, with similar resistant patterns. These cells do exhibit some increased sensitivity due to lower NRF2 expression, potentially caused by side effects of the CRISPR-directed gene editing reaction itself or unknown functions of the Neh2 domain.

Clone 2-23 contains two wildtype alleles and an allele with a two-base pair deletion at the CRISPR cleavage site. This altered allele does not induce exon skipping and the only transcript present is wildtype NRF2. Thus, the response to cisplatin correlates with the profile of the mRNA transcript population. For example, if clone 2-23 generates only wildtype transcript, the cells should behave in a similar fashion to A549 parental cells. Our data fulfills this prediction as this clone appears to be slightly more resistant to cisplatin than wildtype cells.

We expanded this analysis further by carrying out experiments with a second set of clonal cell lines, clones 15, 21, 32, and 33, and the A549 parental cells (Fig. 6B). Each clonal cell line contains a unique composition of mRNA molecules (listed in Fig. 2). Clonal cell lines 33 and 21 have similar responses to cisplatin treatment. The response of clone 33 is predicted because it harbors two transcript populations—one population with exon 4 skipping and the other missing 101 base pairs, consistent with the indel pattern seen in the genomic DNA. Clone 21 contains a 1, 103, and 101 bp deletion in the genomic DNA which is reflected in the mRNA. As seen in the western blot in Fig. 4B, clone 21 does not support detectable NRF2 protein expression, therefore, the loss of NRF2 should sensitize the cells to chemotherapy. As predicted, clone 21 does show increased sensitivity to cisplatin even at lower concentrations. In contrast, clones 15 and 32 display chemoresistance like that seen with the A549 parental cell line.

Figure 6A and B presents a genetically diverse population of cells targeted with CRISPR to disrupt NRF2. Each clonal cell line presents with a unique molecular outcome that dictates its response to chemotherapy. To summarize the differences in sensitivity to chemotherapy because of exon skipping in NRF2, we compiled the data from those clonal cell lines in Fig. 6C along with the complete NRF2 knockout observed in Clone 21 for comparison. As observed in the graph, the pattern in response to increasing concentrations of cisplatin is similar among all four clonal cell lines as compared to the A549 parental cells. This interesting result will be examined in greater detail, correlating genetic rearrangement with phenotypic response such as chemotherapy, immunotherapy, or even radiation therapy. We will continue to follow this line of investigation by targeting additional genes such as EGFR, TP53 and even CDKN2A, although the focus of this paper for the foundational establishment of exon skipping in NRF2 involved a deep analytical examination in A549 and H1703 cells respectively.

## Discussion

As CRISPR-directed gene editing works its way through the clinical trial process [30–32], it is imperative that investigators analyze the spectrum of genetic outcomes generated by this breakthrough genetic technology. In the past, genetic medicines moved forward quickly, and many became aware of the sobering results. While genetic modification holds unbridled potential for patients with cancer and inherited diseases, previous iterations have fallen short of the mark. We are cognizant of these facts and as we advance gene editing as an augmentative therapy for solid tumors, we have purposely taken a reductionist and methodological approach to evaluate the diversity of targeted and edited cancer cell populations at the genotypic, phenotypic, and functional levels.

Our experiments were designed specifically to evaluate how tumor cells would respond to CRISPR/Cas and our results indicate that a heterogeneous population expressing variations of NRF2 is produced. We discovered that exon skipping is not only active in response to CRISPR/Cas DNA cleavage, but in some cases, altered transcripts dominate the population. A major finding of our work is that the presence and position of ESEs should be given serious consideration in the design of gRNAs a fundamental component of the CRISPR/Cas system. In fact, one could argue that disruption of ESEs is more detrimental than the more publicized possibility of off-site mutagenesis. As we have described previously [6], on-site mutagenesis has a direct impact on gene expression in the mis-regulation of control mechanisms.

The results presented in the current manuscript arise from experiments carried out with a series of guide RNAs targeting non-adjacent exons with both single and dual knockout strategies. This matrix workflow was purposely designed so we could ensure that information gained from such studies did not arise simply from a unique site within *NRF2*. Earlier work from our lab has informed combinatorial experimental designs due to the diversity of genetic outcomes from CRISPR/Cas activity on mammalian genes [33–37]. Here, we confirm the existence of this genetic diversity at the RNA level as well, with multiple transcripts arising from the genetically reengineered gene. Furthermore, these transcripts are translated into proteins with altered function. Our preliminary analyses of gene expression changes in these clonal cell lines reveal downregulation and upregulation of genes predicted to be affected by the knockout of NRF2.

Previously, we established that CRISPR-directed knockout of *NRF2* within exon 4 disrupts the nuclear export signal of the intact protein, changing phenotype and accomplishing the desired outcome [6]. While NRF2 is translated into a truncated protein, it cannot penetrate the nucleus and therefore its ability to activate stress response genes is blocked to a large degree. As predicted, genetic rearrangement of *NRF2* significantly lowers resistance to the killing action of various forms of chemotherapy. While this has certainly provided impetus to advance a clinical strategy, the lingering presence of the NRF2 protein, admittedly lacking demonstrable functions, prompted us to look more deeply at the molecular consequences of *NRF2* gene knockout from a transcriptional standpoint.

As part of the detailed analyses, we created a series of clonal isolates that differ in the extent of *NRF2* allelic knockout. One of these clonal isolates illustrates the complexity and diversity of the cellular response to CRISPR-directed gene editing. Clone 1-17 contains a two-base pair deletion at the CRISPR cleavage site across all alleles of *NRF2* in A549 cells. As a result, a mixed population of transcripts is present in the clonal cell population. This mixed population consists of transcripts with the two base pair deletion, as predicted from the genomic information, while the other transcript population is devoid of exons two and three. We confirmed through smFISH that exon skipping NRF2 transcripts are predominately driving phenotypic response and the loss of these exons causes functional consequences (Fig. 6). The absence of exon two and three results in the loss of functional Neh2 and Neh4 domains, respectively. The loss of Neh2 domain is likely not detrimental to the cell because Neh2 encodes the KEAP1 protein binding domain and in the A549 cells, this function is inactive through a mutation in the first Kelch repeat of *KEAP1*. Thus, it is the loss of the Neh4 domain that likely drives the heightened chemo-sensitive phenotype of the cell because it is involved in the transactivation and transcription of downstream target genes of NRF2 [38, 39].

The precision of on-site mutagenesis [33, 37] leading to the creation of altered transcripts is surprising. For example, the loss of two nucleotides in the middle of the fifth ESE in exon 2 (clone 1-17) disrupts the ESE and impacts functionality, whereas the single base pair deletion in clone 2-16 precedes the fourth ESE of exon 2, leaving the ESE intact. Tampering with ESE sites and the surrounding bases sets in motion the skipping of exon 2 in both clones. We have summarized how the subtle changes can evolve altered transcripts in Supplemental Fig. 4.

The generation of transcripts missing individual exons could clearly influence the effectiveness of frontline therapy. Strong evidence for this can be found in data presented in Fig. 5. Here, we establish a correlation between the severity of NRF2 knockout and cell proliferation/toxicity when the targeted cells are challenged by cisplatin. Thus, the diversity of genetic outcomes at both the DNA and RNA level within a targeted population of tumor cells, should be understood to ensure proper dosing of the frontline therapy to achieve maximal effectiveness.

We are not the first to report CRISPR-induced exon skipping. Previous publications include analysis of the activity of a variety of Cas nucleases [40, 41], base editors [42, 43], and CRISPR expression systems [44]. Some of these studies identify novel mRNA isoforms that result in aberrant protein function arising from CRISPR-induced exon skipping [45–49]. As noted by Tuladhar et al. [19], there are other factors that contribute to splicing, such as RNA structural changes. In large part, disruption of the ESE leads to truncated or alternatively spliced transcripts, a phenomenon that we observe in our experiments.

We suggest that investigators undertake a similar detailed analysis of DNA sequence alterations caused by CRISPR/Cas to evaluate how small precise changes created by the design and execution of the gRNA could have dramatic effects on phenotypic outcomes. Our studies reported herein demonstrate the importance of such analyses. For example, it matters which protein domains are eliminated by exon skipping as the absence of certain protein domains can lead to a diversity of responses to chemotherapy, radiation therapy, or even immunotherapy. We do not mean to suggest that CRISPR/Cas is not nor will not be an extraordinarily valuable tool for genetic medicine, but rather urge careful examination of genetic outcomes resulting from the activity of innovative bio-therapeutics early in the pathway of clinical development.

## Supplementary information

Supplemental Figures
