# Supplementary material for: Exon skipping induced by CRISPR-directed gene editing regulates the response to chemotherapy in non-small cell lung carcinoma cells
Source: Gene Ther. 2022 Mar 22;29(6):357–67. doi: 10.1038/s41434-022-00324-7 (PMC9203268; doi:10.1038/s41434-022-00324-7)
Supplement: Supplementary file 1 — Supplemental Figures [file 41434_2022_324_MOESM1_ESM.pdf]

Supplemental Figure 1

A

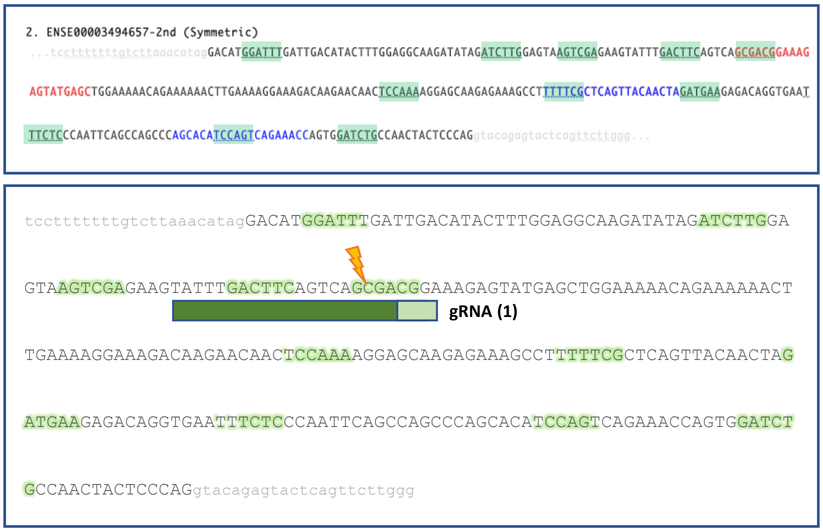

Cell line	Clone	gRNA	gDNA	mRNA
A549	1-17	gRNA (1)	-2<br>-2<br>-2	-2<br>Δex 2 & 3
A549	2-16	gRNA (1)	-1<br>-1<br>-1	-1<br>Δex 2
A549	2-23	gRNA (1)	WT<br>WT<br>-2	WT

B

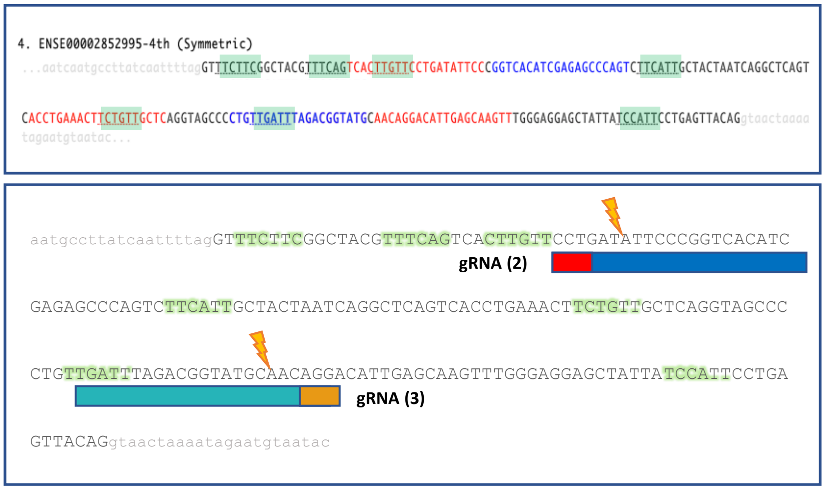

Cell line	Clone	gRNA	gDNA	mRNA
A549	1-40	gRNA (2)	-9<br>-9<br>WT	-9<br>WT
A549	2-11	gRNA (3)	-1<br>-6<br>-10	-1<br>-6<br>-10
A549	33	gRNA (2) & gRNA (3)	-101<br>-101<br>-101	-101<br>Δex 4

Supplemental Figure 1. Relative location of Exonic Splicing Enhancer regions in relation to various CRISPR target sites. The coding region of the *NRF2* gene was analyzed by CRISPinatoR for ESEs (highlighted in green) in exon 2 **(A)** and exon 4 **(B)**. The left panel displays the output of ESEs from CRISPinatoR with the ESEs highlighted in green and the location of each guide RNA used along with each intended cleavage site. The right panel lists a summary of each clonal cell line.

Supplemental Figure 2

A

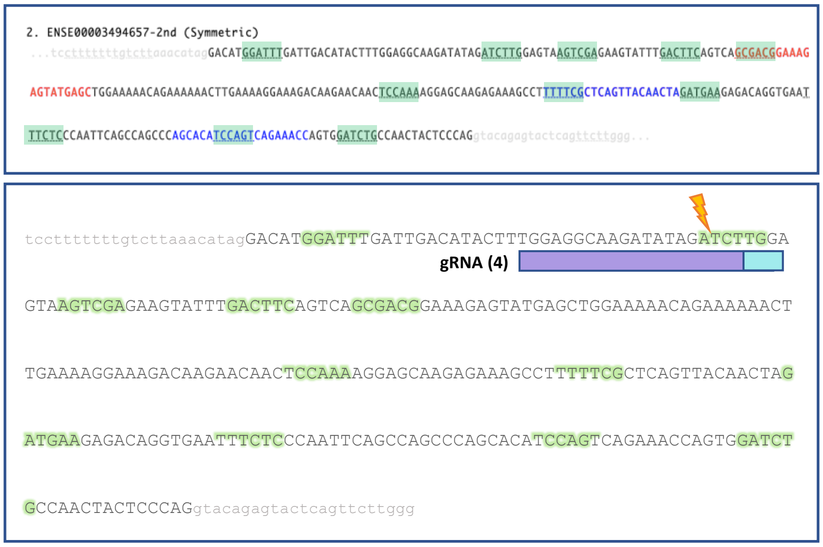

Cell line	Clone	gRNA	gDNA	mRNA
H1703	06	gRNA (4)	-2<br>-2	-2
H1703	32	gRNA (4)	-1<br>-1	-1<br>Δex 2
H1703	43	gRNA (4)	-1<br>-1	-1<br>Δex 2<br>& 3

B

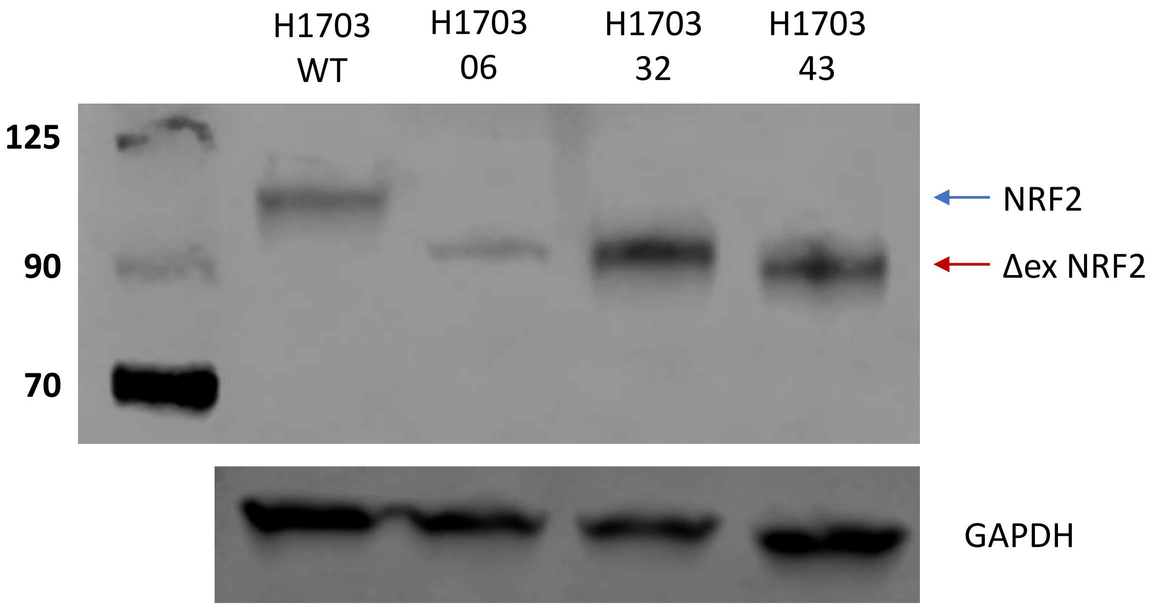

Supplemental Figure 2. (A) Relative location of Exonic Splicing Enhancer regions in relation to various CRISPR target sites. The coding region of the *NRF2* gene was analyzed by CRISPinatoR for ESEs (highlighted in green). A second cell line, H1703, was also analyzed using gRNA (4) targeting exon 2 which resulted in similar exon skipping patterns seen in the A549 clonal cell lines. **(B)** Representative western blot analysis of CRISPR-engineered H1703 clonal cell lines. Clonal cells were harvested for western blot analysis using an antibody directed against NRF2 and GAPDH was used as a loading control.

Supplemental Figure 3

Cell line/Clone	gDNA	mRNA	Protein
**EXON 4<br>gRNA (2) & (3)**	1) WT	1) WT	1) WT
**A549 C15**	1) -1<br>2) -103<br>3) -102	1) -1<br>2) -103<br>3) -102	1) No protein<br>2) No protein<br>3) Protein
**A549 C21**	1) -1<br>2) -103<br>3) -101	1) -1<br>2) -103<br>3) -101	1) No protein<br>2) No protein<br>3) No protein
**A549 C32**	1) -103<br>2) -104<br>3) -102	1) -103<br>2) -104<br>3) -102	1) No protein<br>2) Protein (?)<br>3) Protein
**A549 C33**	1) -101<br>2) -101<br>3) -101	1) -101<br>2) Δex 4	1) No protein<br>2) Protein

Supplemental Figure 3. Genomic, transcript and protein summary of A549 clonal cell lines. The fourth column lists which transcript is likely being translated to produce protein based on the information gained from clone 21. Clone 21 has a mixed population of transcripts, -1, -103 and -101, however, none of the transcripts are translated to protein based on the western blot. What we know about the protein expression pattern of clone 21 can be translated to the rest of the clones. Based on the western blot, clone 21 does not produce protein so all three transcripts are being degraded. This means the -1 and -103 bp transcripts also seen in clone 15 are being degraded, and the -102 bp transcript is likely driving protein expression. The -102 bp transcript is also seen in clone 32 along with a -104 bp transcript (which may or may not produce protein) however, based on the previous knowledge, the -103bp transcript is being degraded. On the western blot, both clone 15 and clone 32 have similar blotting patterns, which again points to the -102 bp transcript driving protein expression.

Supplemental Figure 4

Clone 1-17

2bp deletion

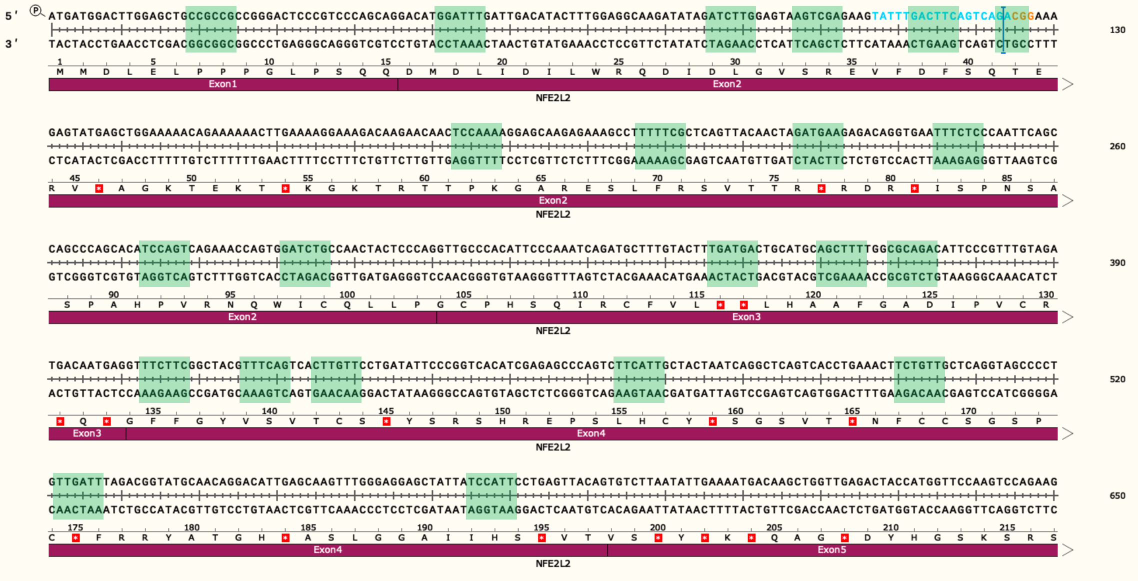

Clone 2-16

1bp deletion

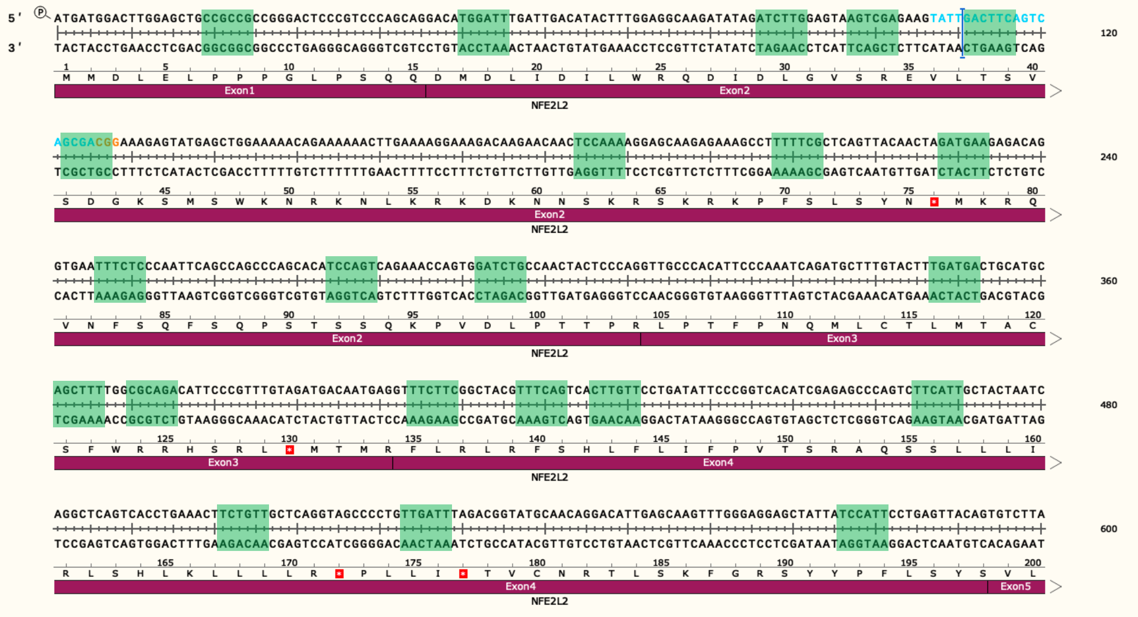

Supplemental Figure 4. Schematic diagram of indel-specific NRF2 transcript in the A549 clonal cell lines. The top panel presents the altered transcript that is created in clone 1-17 as a result of the two base pair deletion indicated by the vertical line. The bottom panel presents the altered transcript that is created in clone 2-16 as a result of the single base pair deletion indicated by the vertical line. In both panels, the guide RNA sequence is in blue and PAM in orange. Each ESE is highlighted in green. The red asterisks indicate new stop codons formed as a result of the indel frameshift.
